# *In Vitro* Models in Biocompatibility Assessment for Biomedical-Grade Chitosan Derivatives in Wound Management

**DOI:** 10.3390/ijms10031300

**Published:** 2009-03-18

**Authors:** Lim Chin Keong, Ahmad Sukari Halim

**Affiliations:** Reconstructive Sciences Unit, School of Medical Sciences, Universiti Sains Malaysia, Kubang Kerian, Kelantan, Malaysia

**Keywords:** Biopolymer, chitosan, biocompatibility, biomedical-grade chitosan, *in vitro* toxicity examinations, cytocompatibility, genotoxicity, skin pro-inflammatory cytokine expression

## Abstract

One of the ultimate goals of wound healing research is to find effective healing techniques that utilize the regeneration of similar tissues. This involves the modification of various wound dressing biomaterials for proper wound management. The biopolymer chitosan (β-1,4-D-glucosamine) has natural biocompatibility and biodegradability that render it suitable for wound management. By definition, a biocompatible biomaterial does not have toxic or injurious effects on biological systems. Chemical and physical modifications of chitosan influence its biocompatibility and biodegradability to an uncertain degree. Hence, the modified biomedical-grade of chitosan derivatives should be pre-examined *in vitro* in order to produce high-quality, biocompatible dressings. *In vitro* toxicity examinations are more favorable than those performed *in vivo,* as the results are more reproducible and predictive. In this paper, basic *in vitro* tools were used to evaluate cellular and molecular responses with regard to the biocompatibility of biomedical-grade chitosan. Three paramount experimental parameters of biocompatibility *in vitro* namely cytocompatibility, genotoxicity and skin pro-inflammatory cytokine expression, were generally reviewed for biomedical-grade chitosan as wound dressing.

## Contents

1. Introduction
2. Chitosan derivatives in wound management
3. Biocompatibility *in vitro* of biomedical-grade chitosan derivatives
4. Biocompatibility *in vitro* experimental tools
  4.1. Cytocompatibility assessment in vitro
  4.2. Genotoxicity assessment in vitro
  4.3. Assessment of human skin pro-inflammatory cytokines
5. Future considerations and conclusion

Acknowledgments

References

## 1. Introduction

This paper generally reviews the literature on the *in vitro* models for biocompatibility assessment of chitosan derivatives for use in human wound management. Wound healing is a process of restoration that is necessary for tissue repair, typically comprising a continuous sequence of inflammation and repair in which epithelial, endothelial, inflammatory cells, platelets and fibroblasts briefly interact to resume their normal functions. This healing process consists of four different and overlapping phases, namely: inflammation, granulation tissue formation, matrix remodeling and reepithelialization [1]. The orderly sequence of healing events is performed and regulated by cytokines and growth factors [2]. One of the ultimate goals in wound healing research involves finding ways for humans to heal *via* the regeneration of similar tissues. This could be accomplished *via* the invention and modification of a variety of dressing materials to facilitate proper wound management.

Chitosan is a partially deacetylated derivative of natural chitin, a primary structural polymer in arthropod exoskeletons. One of the present trends in biomedical research requires materials that are derived from nature as natural materials have been shown to exhibit better biocompatibility with humans. For example, chitosan’s monomeric unit, *N*-acetylglucosamine, occurs in hyaluronic acid, an extracellular macromolecule that is important in wound repair. It has been considered as a candidate natural polymer for the scaffold since chitosan shows several advantages over other synthetic biomaterials and easy manipulation of stable porous structure by lyophilization. Studies on chitosan have been intensified since 1990 due to its low cytotoxicity, antimicrobial activity and excellent biodegradable properties in the human body, where these have contributed to the better biocompatibility of chitosan [3–6]. Additionally, the *N*-acetylglucosamine moiety in chitosan is structurally similar to glycosaminoglycans (GAGs), heparin, chondroitin sulphate and hyaluronic acid in which they are biocompatible, and hold the specific interactions with various growth factors, receptors and adhesion proteins besides being the biologically important mucopolysaccharides and in all mammals. Therefore, the analogous structure in chitosan may also exert similar bioactivity and biocompatibility [7–8].

The potential of chitosan stems from its cationic nature and high charge density in solution. An effective approach for developing a clinically applicable chitosan is to modify the surface of the material that already has excellent biofunctionality and bulk properties. Widely used surface modification techniques include coating, oxidation by low-temperature plasma and surfactant addition in order to blend with various derivatives. However, blending technologies with various additives may cause cytotoxicity. Hence, the modified biomedical-grade of chitosan with various derivatives should be submitted for biocompatibility testing to promote the engineering of high-quality, biocompatibility for human wound management.

## 2. Chitosan Derivatives in Wound Management

Chitosan (β-1,4-D-glucosamine) is a deacetylated form of chitin with excellent biological properties in wound management [9–11]. Altering the physical and chemical properties of the chitosan in order to improve its medicinal quality will also influence its biocompatibility. Chitosan oligosaccharides with a degree of polymerization (DP) between 6 and 8 were found to have antitumor activity [12]. The high percentage of nitrogen (6.89%) found in chitosan has made it a useful chelating agent [13]. Their unique properties include polyoxysalt formation, as well as the ability to form films and chelate metal ions [14].

The excellent biological properties of chitosan can be potentially improved with a variety of additional chemicals such as polyethylene glycol and carboxymethyl, *N*-acyl groups in order to produce biocompatible chitosan derivatives for use as wound dressings [15]. Chitosan’s positive surface charge enables it to effectively support cell growth [16]. Chitosan-gelatin sponge wound dressing had demonstrated a superior antibacterial effect, as mediated by vacuum driness on *Escherichia coli* K88, in comparison to that of penicillin. Furthermore, chitosan gelatin-sponge wound dressing displayed a superior healing effect to that of 0.2% (v/v) ethacridine. Additionally, chitosan-gelatin sponge allowed the wound site to contract markedly and shortened the wound healing time, as compared with sterile Vaseline gauze [17]. Clinical assessment was performed using a Hyphecan cap (1–4,2-acetamido-deoxy-β-D-glucan), as a biological fingertip dressing and was proven to be effective in achieving a good functional and cosmetic result within a relatively short time, aside from being comfortable during conventional dressing changes [9].

## 3. Biocompatibility *In Vitro* of Biomedical-Grade Chitosan Derivatives

Biocompatibility of a biomaterial refers to the extent to which the material does not have toxic or injurious effects on biological systems. In the pre-commercialization steps of a biomedical-grade chitosan derivative as wound dressing, it is of great importance that the dressing be submitted to a number of pre-toxicity tests, *in vitro* or *in vivo.* Chitosan is a cationic polymer having an amino group in its chemical structure. Anti-tumor, anti-microbial and anti-inflammatory can be largely attributed to the cationic structure. Lee *et al.* [18] performed trypan blue dye exclusion cytotoxicity assays on tumor cell lines, after increased positive charge density of chitosan by dialkylaminoalkylation and reductive amination followed by quaternization. Cytotoxicity of chitosan with various degrees of *N-*deacetylation to form aminoethyl-chitosan, dimetylaminoethyl-chitosan and diethylaminoethyl-chitosan, was examined only with 3-(4,5-dimethylthiazol-2-yl)-2,5-diphenyl tetrazolium bromide (MTT) on tumor cell lines and concluded that cytotoxic effect may be caused by cationic charge of the chitosan derivatives [19]. Amino groups of chitosan attached with biologically active moieties such as vanillin, *p*-hydroxybenzaldehyde, *p*-chlorobenzaldehyde, anisaldehyde, methyl 4-hydroxybenzoate, methyl 2,4-dihydroxybenzoate, propyl 3,4,5-trihydroxybenzoate and 2-hydroxymethylbenzoate can promote anti-microbial ability [20].

A thorough understanding of cell and proteins interactions with artificial surfaces is of importance to design suitable implant surfaces and substrates. The surface properties of newly synthesized biomedical-grade chitosan derivatives, including surface composition, wettability, domain composition, surface oxidation, surface charge and morphology, may influence protein adsorption and subsequently, the cellular responses to biomaterial implants. Though it has once been suggested that surfaces modified with polyethylene glycol (PEG) may limit protein adsorption and cell adhesion, the results for PEG blended with chitosan increased both the adsorption and the cell adhesion [21]. Zhang *et al*. [22] examined the biocompatibility of newly synthesized chitosan-PEG by growing and observing the morphological changes of 3T3 embryonic mouse fibroblasts and osteogenesis nodule cell suspension in culture. Cytotoxicity of chitosan in three dimensional fiber meshes was well determined with only scanning electron microscopy (SEM) and cell morphological observation on the use of mouse lung fibroblast (L929) [23]. Recent studies have been focused on the development of antibacterial surfaces of biomedical-grade chitosan derivatives to attain high functionality and high-value products. Huh *et al.* [24] prepared chitosan-grafted poly(ethylene terepthalate) (PET) and quaternized chitosan-grafted PET, both showed high growth inhibition in the range of 75% to 86% and still retained 48% to 58% bacterial growth inhibition after laundering. But, higher antibacterial activity of a wound dressing can retard the cell growth in culture. Laminin is known to be involved in metastasis of tumor cells. A peptide containing the Tyr-Ile-Gly-Ser-Arg (YIGSR) sequence, corresponding to a partial sequence of laminin, inhibited angiogenesis and therefore depressed tumor growth. Nishiyama *et al*. [25] prepared YIGSR-chitosan conjugate and proved to have higher inhibitory activity against experimental lung metastasis of B16BL6 melanoma cells in mice than did the parent peptide. According to Koo *et al*. [26], water-soluble chitosan (WSC) significantly protected from serum starvation-induced cellular rounding up and from serum starvation-induced cell death as tested by flow-cytometry. WSC also protected from serum starvation-induced p53 activation as determined by the Western blot technique. From these results, it appears that WSC may prevent serum starvation-induced apoptosis of human astrocytes *via* p53 inactivation. Cross-linking will increase the toxicity of chitosan scaffold leachate to fibroblast cells. However, the chitosan crosslinked with dimethyl 3-3,dithio-bis-propionimidate (DTBP) permitted cell proliferation after three days in culture [27]. Denuziere *et al*. [28] examined the cytocompatibility of films of chitosan and chitosan associated with glycosaminoglycans (GAGs) with human skin keratinocytes and found that the cell growth was consistent approximately 60% of that of controls. Highly deacetylated chitosans can modulate human skin cell mitogenesis *in vitro*. Analysis of their effects on cells in culture may be useful as a screen for their potential activity *in vivo* as wound healing agents. But, it is important to select appropriate strains of cells for use in the *in vitro* biocompatibility screening [29]. Still, most of the biocompatibility *in vitro* screenings to date for chitosan derivatives confined at cellular responses and reactions, namely at the cytocompatibility level [30–34].

## 4. Biocompatibility *In Vitro* Experimental Tools

*In vitro* models for testing the biocompatibility of biodegradable chitosan and their derivatives are very useful to characterize the hidden toxic effects of leachable materials or derivatives, such as residual monomers, catalysts as well as other polymer erosion related properties, such as chemical compositions, molecular weight, polydispersity, degradation rate and so forth. Cytotoxicity assays measure only finite effects on cells during the first 12 to 24 hours after exposure to toxic substances. The host cells either recover from or succumb to their chemical injury. However, many biological reactions *in vivo* are not simply cytotoxic and are propagated beyond 24 hours. Examples are improper cells programmed apoptosis and necrosis due to DNA damage; inflammatory and immune reactions that all resulted from non-biocompatible chitosan derivatives.

*In vitro* toxicity examinations are preferable to *in vivo* tests since the newly developed dressing materials can be examined outside the body and the results are more reproducible, facilitating clinical evaluation of a medicinal product that can be replicated and estimated [35–36]. It is necessary to determine the biocompatibility of any biomaterial in order to evaluate its strength, esthetics and feasibility for clinical manipulation. Thus, preliminary *in vitro* tests are continually being carried out to screen and characterize the potentially harmful effects of a dressing material before it is commercially used on humans [15]. However, *in vitro* tests may not fully capture the *in vivo* situation; differences in sensitivities have been observed between primary and established cell lines [37–38]. Current regulations in accordance with the United States Food and Drug Administration (FDA), the International Organization for Standardization (ISO) and the Japanese Ministry of Health and Welfare (JMHW) require that manufacturers conduct adequate safety testing of their finished devices through pre-clinical and clinical trials as part of the regulatory clearance process. Toxicity induced by a chemical substance or drug can limit the dose and duration of treatment, adversely affect patients’ quality of life or even be life threatening. Side effects together with tissue toxicity will lead to long-term effects such as alteration to the immune system and development of malignancies, probably due to the genetic damage induced by a certain kind of drug treatment [39]. Hence, biocompatibility assessment *in vitro* is necessary to evaluate cellular and molecular responses to the newly developed biomedical-grade chitosan derivatives.

### 4.1. Cytocompatibility assessment in vitro

There are a number of assays which are capable of evaluating the activation of biological processes (*e.g*. inflammation, immune reactions and mutagenesis), which require longer periods of tissue reaction to materials, as compared with cytotoxic reactions. Traditional assays have measured cytotoxicity using either an end-stage event (*e.g.* permeability of cytoplasmic membranes of dead and dying cells, or some metabolic parameters such as cell division or enzymatic reaction) [40]. Cytotoxicity assays are commonly used to measure the effects on cells during exposure to toxic substances at multiple concentrations and time course parameters. The colorimetric 3-(4,5-dimethylthiazol-2-yl)-2,5-diphenyl tetrazolium bromide (MTT) assay is a simple assay which is used to identify detrimental intracellular effects on mitochondria and metabolic activity, based on the selective ability of viable cells to reduce tetrazolium bromide into purple formazan crystals, which are only soluble in organic solvents. The extent of formazan crystal formation depends on intact metabolic activity and is frequently used for cytotoxicity screening [41]. The MTT assessment to the cytocompatibility was adequately good and the results were easily interpreted, besides being cost effective [32–36].

In our previous research paper [42], *in vitro* biocompatibility evaluation of biomedical-grade oligochitosan (O-C) and *N,O*-carboxymethyl-chitosan (NO-CMC) derivatives (O-C 1%, O-C 5%, NO-CMC 1% and NO-CMC 5%) correlated well with *in vivo* results in which O-C 1% being the most cytocompatible. The experiment was carried out as described by the International Standards Organization (ISO) [43], with some modifications. One of the most basic and important parameters in the *in vitro* measurement of a medicinal product’s biocompatibility is cytocompatibility, which measures the qualitative and quantitative aspects of the impact of a medicinal product with regard to the viability of target cultured cells. Biomedical-grade chitosan derivatives are to be used primarily in human wound management. Thus, the target cultured cells in our research are primary normal human skin epidermal keratinocytes (pNHEK) and primary normal human skin dermal fibroblasts (pNHDF), which are primarily found in the skin epidermis and dermis respectively. Several transformed keratinocyte culture models have been used to study carcinogenesis, differentiation, apoptosis and cell cycle regulation [44]. Our focus here is on normal (non-transformed) keratinocyte culture models because cutaneous toxicity testing of new drugs, cosmetic products and other chemicals requires phenotypically normal cell systems [45]. This is because the loss of differentiated function in transformed cell types undoubtedly confounds interpretation of the results [46]. Some have suggested that a better correlation of *in vitro* toxicity testing with irritation potential *in vivo* may be obtained by using normal keratinocyte culture models as opposed to transformed cell lines [47]. Furthermore, various *in vitro* systems are used for cutaneous toxicity testing, ranging from submerged keratinocyte and liquid-air interface skin cultures to three-dimensional reconstructed skin models. However, our focus here is on submerged monolayer epidermal keratinocyte culture models. The primary reason for this focus is a key concept in support of incorporating keratinocyte culture models into a battery of cutaneous toxicity tests, namely keratinocytes make up the bulk (greater than 90%) of epidermal tissue mass [48], and viable keratinocytes (not exposed directly to air) participate in the conversion of environmental stimuli into biological signals that activate the inflammatory and hyper-proliferative responses of the skin [49]. Therefore, submerged cultures of epidermal keratinocytes model a major component of the target epidermal tissue, allowing an assessment of the effects of drugs or chemicals on the skin. The use of low-density polyethylene (LDPE) as negative control is also recommended in the ISO cytotoxicity guidelines. LDPE is widely used in the manufacture of petri dishes and culture flasks, where LDPE is proven to be non-toxic to cultured cells. The comparison between control without any treatment and negative control (with LDPE) indicated in all tests that the cells in the negative control condition were slightly lesser than the untreated cells. This effect might be due to the weight impact of LDPE on cultured cells. LDPE might slightly inhibit the circulation of the enriched medium from the upper level to the lower level of a culture well. The net cytotoxicity effect of the biomedical-grade chitosan on cultured cells can be evaluated more accurately if they are compared to the negative control in order to eliminate the impact on physical weight from the calculation.

### 4.2. Genotoxicity assessment in vitro

Although the measures of a medical product's biocompatibility have largely been reported in terms of irritation, sensitization, and systemic toxicity, there is a growing concern that devices or biomaterial extracts may exert some genotoxic effects, even though they may be cytocompatible in cytocompatibility *in vitro* assessment. Thus, any attempts to assess the safety of a device intended for intimate body contact or permanent implantation would be incomplete without testing for the presence of toxins that might exert an effect on the genetic material of cells. Genotoxic effects will eventually lead to abnormal and reduced cell growth, even if the cells initially appear cytocompatible. In its set of standards for the biological evaluation of medical devices, the ISO has outlined the need for such genotoxicity testing in ISO 10993-3 [50]. A number of techniques for detecting deoxyribonucleic acid (DNA) damage as opposed to the biological results (*e.g*. Ames test, micronuclei, mutations, structural chromosomal aberrations) [51–54] of DNA damage have been used long time ago to identify substances with genotoxic activity. According to Muramatsu *et al*. [55], they did not detect any genotoxicity performed *via* Ames test and chromosome aberration assay, on Chinese hamster lung fibroblasts with 5mg/mL (maximum concentration) of beta-tricalcium phosphate/carboxymethyl-chitin. Abou Sereih *et al*. [56] reported that their chitosan with concentrations 3.00 and 4.50 mg/mL have induced mutation on *Trichoderma*. However, those techniques have some shortcomings (*e.g*. time investment, requirement for proliferating cell populations) [57–60].

Until recently, a more useful approach for assessing DNA damage has been the single-cell gel electrophoresis (SCGE) or Comet assay. The term “Comet” is used to denote the individual cell DNA migration patterns produced. Ostling and Johanson were the first to develop a microgel electrophoresis technique for detecting DNA damage at the single-cell level [61]. For this technique, cells were embedded in agarose and placed on a microscope slide. The cells were lysed by detergents and high salt concentration to liberate the DNA. The liberated DNA was then electrophoresed under neutral conditions. Therefore, cells with an increased frequency of DNA double strand breaks (DSB) displayed increased migration of DNA toward the anode. The migrating DNA was then assessed quantitatively by staining with ethidium bromide using a microscope photometer. The neutral conditions used greatly limited the general utility of the assay for detecting the DSB of a cell. Subsequently, the microgel technique was introduced, which involved the use of electrophoresis under alkaline (pH >13) conditions for detecting DNA single strand breaks (SSB) and alkali-labile sites (ALS) in a single cell [62]. Consequently, alkaline electrophoresis offers enhanced sensitivity for identifying various unexpected genotoxic agents that tend to induce more SSB and ALS than DSB. The Comet assay requires only a few cells per assay, and it is also cost effective [63]. Many excellent reviews on the use of the Comet assay used aquatic animals to assess DNA damage caused by genotoxicants [64–66]. In addition, production of DNA strand breaks correlates well with the mutagenic and carcinogenic properties of environmental pollutants [65]. Therefore, application of comet assay to evaluate the genotoxicity of various newly modified chitosan derivatives can be another approach.

### 4.3. Assessment of human skin pro-inflammatory cytokines

There are limited studies on the use of skin pro-inflammatory cytokines to assess the biocompatibility of various chitosan derivatives to date. Cytokines are low molecular weight glycoproteins produced by immune as well as non-immune cells. Their roles in regulating immunologic responses, hematopoietic development, and cell-to-cell communication, as well as host responses to infectious agents and inflammatory stimuli, have been widely investigated. Cytokines mediate interactions between various cells and their dysregulated production may lead to disease pathogenesis [67]. Under normal circumstances, cytokines are not detectable at low levels in body fluids or in tissues. Therefore, their elevated levels of expression could indicate activation of cytokine pathways associated with inflammation or disease progression. All cytokines are pleiotropic: they are able to interact with a variety of cellular targets via specific receptors expressed on the surface of target cells. The binding of cytokines to cell surface receptors will initiate RNA signaling and protein synthesis [68]. Cytokines function as networks or cascades of interacting cytokines, where they are able to induce the expression of other cytokines by regulating their own production through autocrine, juxtacrine or paracrine pathways in response to micro-environmental stimuli. Keratinocytes are able to secrete a number of inflammatory cytokines [*e.g.* interleukin 1α(IL-1α), tumor necrosis factor-α (TNF-α)] and chemotactic cytokines [*e.g.* interleukin-8 (IL-8)] when they come in contact with irritants [69]. The changes in cytokine levels could potentially be used as biomarkers of cutaneous vesicant injury [70]. Research on toxicity and inflammation in normal human epidermal keratinocyte (NHEK) cultures in accordance with the use of jet fuel showed similarities between porcine keratinocytes and NHEK in terms of TNF-α and IL-8 expression [71]. Another experiment that demonstrated the chemical irritants induced the production of IL-1, TNF-α and IL-8 [72]. These findings indicate that the secretion of pro-inflammatory and chemotactic cytokines may provide a useful biomarker for determining the toxicity level of irritants on human skin.

One study has suggested that environmental pollutants such as diesel exhaust particles and formaldehyde affect the ability of human skin keratinocytes to release pro-inflammatory cytokines (IL-1α, IL-8 and TNF-α) [73]. It has been reported that IL-8 production by keratinocytes is under the control of protein kinase C [74] and that protein kinase C signaling pathways are altered in keratinocytes from patients with psoriasis [75]. Therefore, by quantitatively examining the amount (pg/ml) of human IL-1α, IL-8 and TNF-α secreted from treated pNHEK cultures, using the enzyme-linked immunosorbent assay (ELISA), the *in vitro* biocompatibility of chitosan derivatives can be evaluated in more detail.

## 5. Future Considerations and Conclusions

Chitosan and its various derivatives have a wide range of applications. They may be employed to solve numerous problems in environmental and biomedical engineering. For example, hydroxymethyl chitin and water-soluble chitosan derivatives are useful for anionic waste streams. While chitosan *N-*benzylsulfonate derivatives can be used as sorbents for removal of metal ions in an acidic medium. Deacetylated chitosan can be easily molded into various forms and their derivatives are digested *in vivo* by lysozomal enzymes. Therefore, it appears that chitosan derivatives can be used as a carrier of a variety of drugs for controlled release applications. Chitosan is useful to be a bio-scaffold for tissue engineering especially for skin and bone. Chitosan in the form of porous structure can serve as a template for bone osteoblasts or skin fibroblasts and keratinocytes where the cells can grow within the scaffold and form accordingly to the shape of the scaffold. For example, chitosan porous structure in a thin-sheet alike can represent a scaffold for skin where skin fibroblasts and keratinocytes grow to form a basic skin substitutes *in vitro*. Furthermore, chitosan possesses all the characteristics required for an ideal contact lens such as optical clarity, mechanical stability, sufficient optical correction, gas permeability, wettability and immunological compatibility. Recently, the transdermal absorption promoting characteristics of chitosan have been explored for nasal and oral delivery of polar drugs to include peptides and proteins and for vaccine delivery. These properties have enabled the chitosan together with appropriate derivatives to be a promising biomaterial for pharmaceutical industry and the other applications.

For the establishing of *in vitro* biocompatibility models, the comparison with experimental animals using *in vivo* toxicity studies is also very useful. However, it is unlikely that the toxicity studies in experimental animals can be completely replaced, since biocompatibility of biomedical-grade chitosan is a complex phenomenon encompassing amongst other immune reactions and interactions with various inflammatory cells. Furthermore, interpretative problems occur because tissue reactions to newly synthesized biomedical-grade chitosan is very complicated that leads to the difficulty to separate between the cytotoxicity and differentiation of replacement skin tissues from inflammatory and immune reactions of the surrounding connective tissue *in vivo*. However, by correlating the biocompatibility results both from *in vitro* and *in vivo*, we can be more assured with the safety use of newly synthesized biomedical-grade chitosan on the human, although the exact results would be after clinical application on human. Biocompatibility *in vitro* is useful to characterize cytotoxic effects of leachable materials, such as residual monomers, catalysts as well as polymer erosion and related factors (e.g. composition, molecular weight and polydispersity) which are suitable to monitor the quality and purity of the biodegradable biomedical-grade chitosan. Besides, *in vitro* model also enables the study of single cell proliferation and invasion pattern within a porous structured biomedical-grade chitosan in which *in vivo* model may be less efficient in the situation. Moreover, by analyzing the cells invasion pattern within a bio-scaffold or porous biomedical-grade chitosan, not only can produce cytotoxicity evaluation but in the meanwhile the structure of the scaffold can be well defined and engineered. Advances in technology and the understanding of biology, chemistry and related fields will inevitably lead to advances of the present *in vitro* biocompatibility models as well as to new models. Recently, improvement in the *in vitro* biocompatibility has been on the use of coculture model especially skin tissues. This model mainly serves to evaluate a more complicated molecular responses and cascades provoked by test substances. Additionally, the application of human-skin like tissue model such as Epiderm^™^ skin model has also been implemented to further resemble human *in vivo* situation for the studies such as *in vitro* skin irritation, dermal corrosion, dermal phototoxicity, percutaneous absorption, transdermal drug delivery skin inflammation and skin psoriasis research. However, it is not cost-effective if only cytotoxicity to be performed.

In the biocompatibility assessment of any newly developed biomaterial, *in vitro* tests should be conducted prior to *in vivo* tests in order to minimize the risk to humans and animals. The study of biocompatibility *in vitro* enables the detailed evaluation of not only cellular, but also molecular responses. Biomedical-grade chitosan can be enriched with various derivatives to achieve the desired or proposed functionality. However, derivatives enrichment may trigger different responses at cellular and molecular level. There could be insufficient proof to ensure the complete biocompatibility *in vitro* if one performs only cell viability because biomedical-grade chitosan derivatives may provoke genotoxic responses which will eventually lead to cell death. Thus, *in vitro* biocompatibility assessment for wound management must consider cytocompatibility, genotoxicity and related pro-inflammatory cytokines expression for better *in vitro* biocompatibility evaluation.
